# Supplementary material for: Growth phase estimation for abundant bacterial populations sampled longitudinally from human stool metagenomes
Source: Nat Commun. 2023 Sep 14;14:5682. doi: 10.1038/s41467-023-41424-1 (PMC10502120; doi:10.1038/s41467-023-41424-1)
Supplement: Supplementary file 1 — Supplementary Information [file 41467_2023_41424_MOESM1_ESM.pdf]

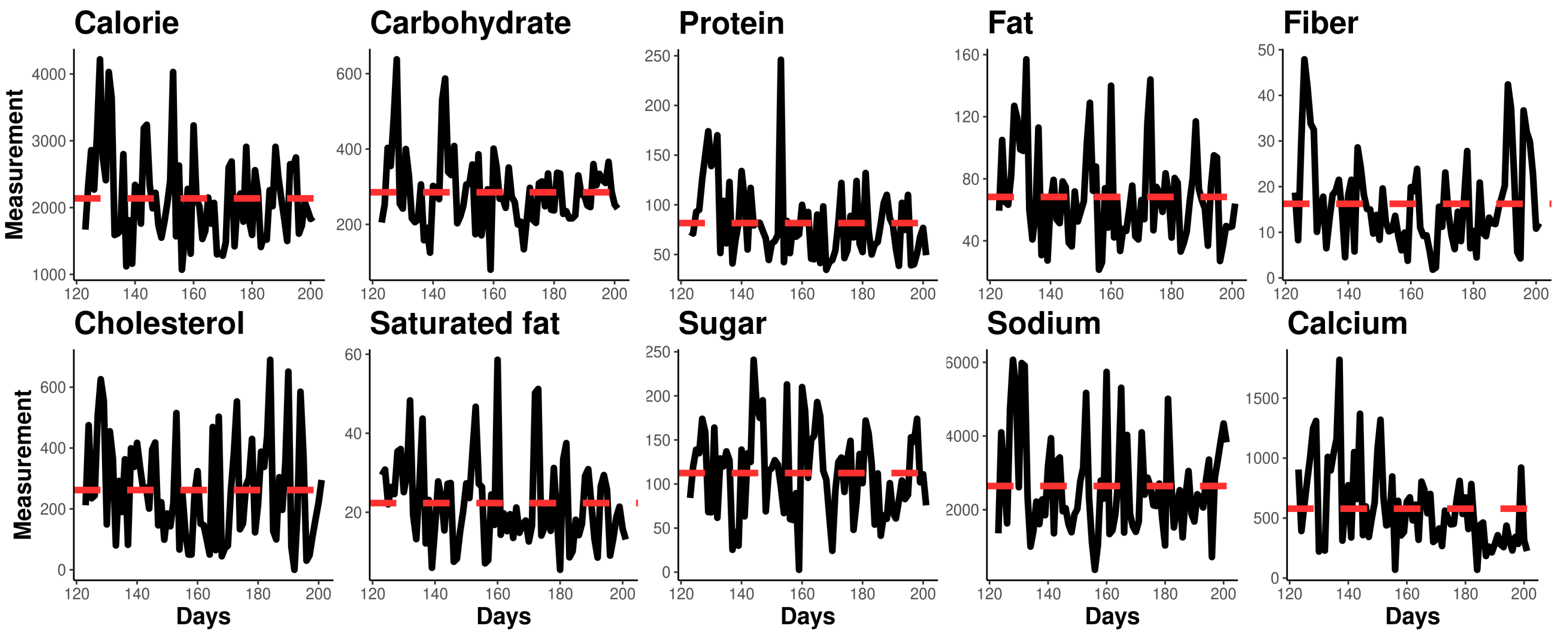

Figure S1. Lack of autocorrelation detected in most daily macronutrient intake variables. Daily measurements of nutrient intake were downloaded from David et al. [1]. Post-travel time points are shown. Autocorrelation was tested using the augmented Dickey-Fuller test (i.e., $p < 0.1$ indicates significant stationarity of a dietary variable). For each nutrient, p-values are reported for the recordings post-travel period of the subject. Calorie ($p = 0.0341$), carbohydrate ($p = 0.0144$), protein ($p = 0.0314$), fat ($p = 0.0172$), fiber ($p = 0.0369$), cholesterol ($p = 0.0534$), saturated fat ($p < 0.01$), sugar ($p = 0.0123$), sodium ($p = 0.0341$), calcium ($p < 0.01$). Dotted red lines show the mean measurement values.

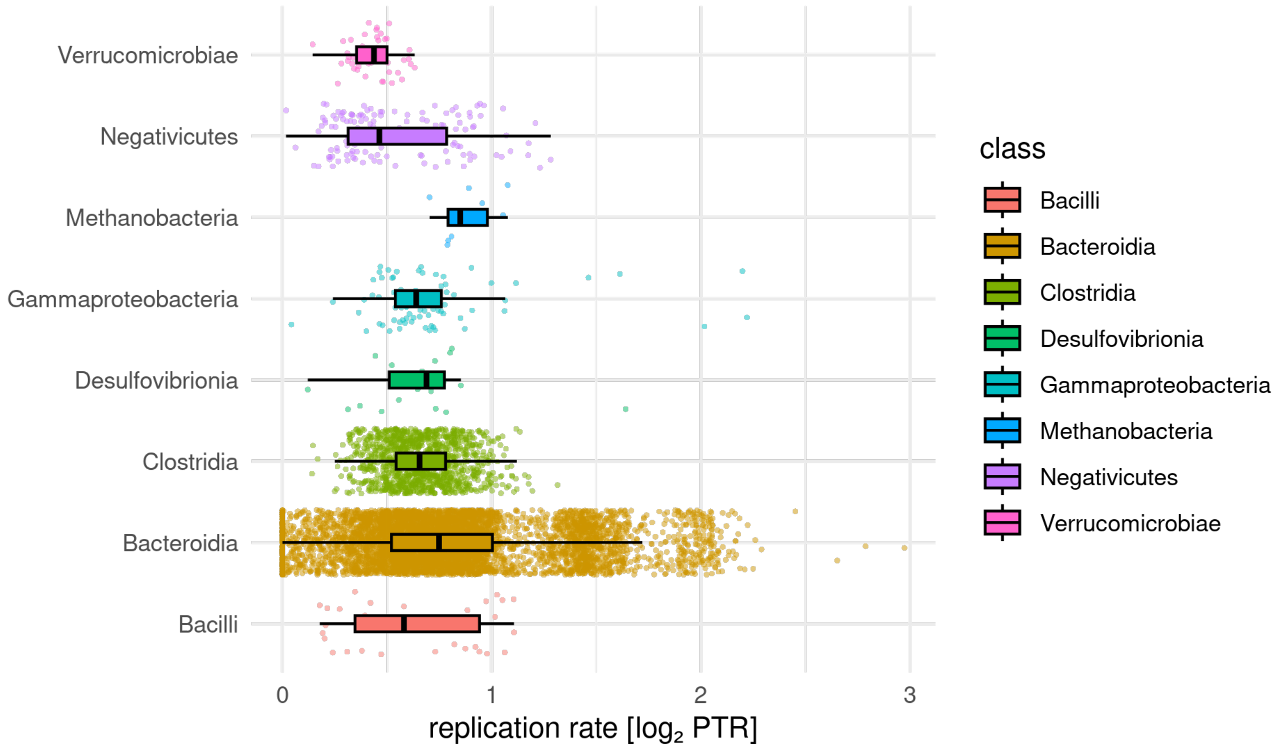

Figure S2. Distributions of $\log_2$(PTR) values across 84 BIO-ML donors, broken down by phylogenetic class. We see a fairly wide range of $\log_2$(PTRs) within each taxonomic class. The median $\log_2$(PTR) across classes varies between ~0.45 and ~0.75. In a linear regression model, controlling from taxonomic group as a covariate, we see a significant positive association between $\log_2$(PTRs) and CLR abundances at the class level ($\beta = 0.0612$, $p = 8.359e^{-60}$; number (n) for each class: Verrucomicrobiae = 44, Negativicutes = 136, Methanobacteria = 8, Gammaproteobacteria = 77, Desulfovibrionia = 20, Clostridia = 1211, Bacteroidia = 5487, Bacilli = 33) . This positive taxonomy-controlled association is preserved at the species level ($\beta = 0.0101$, $p = 0.0006$, total number of species count = 7016).

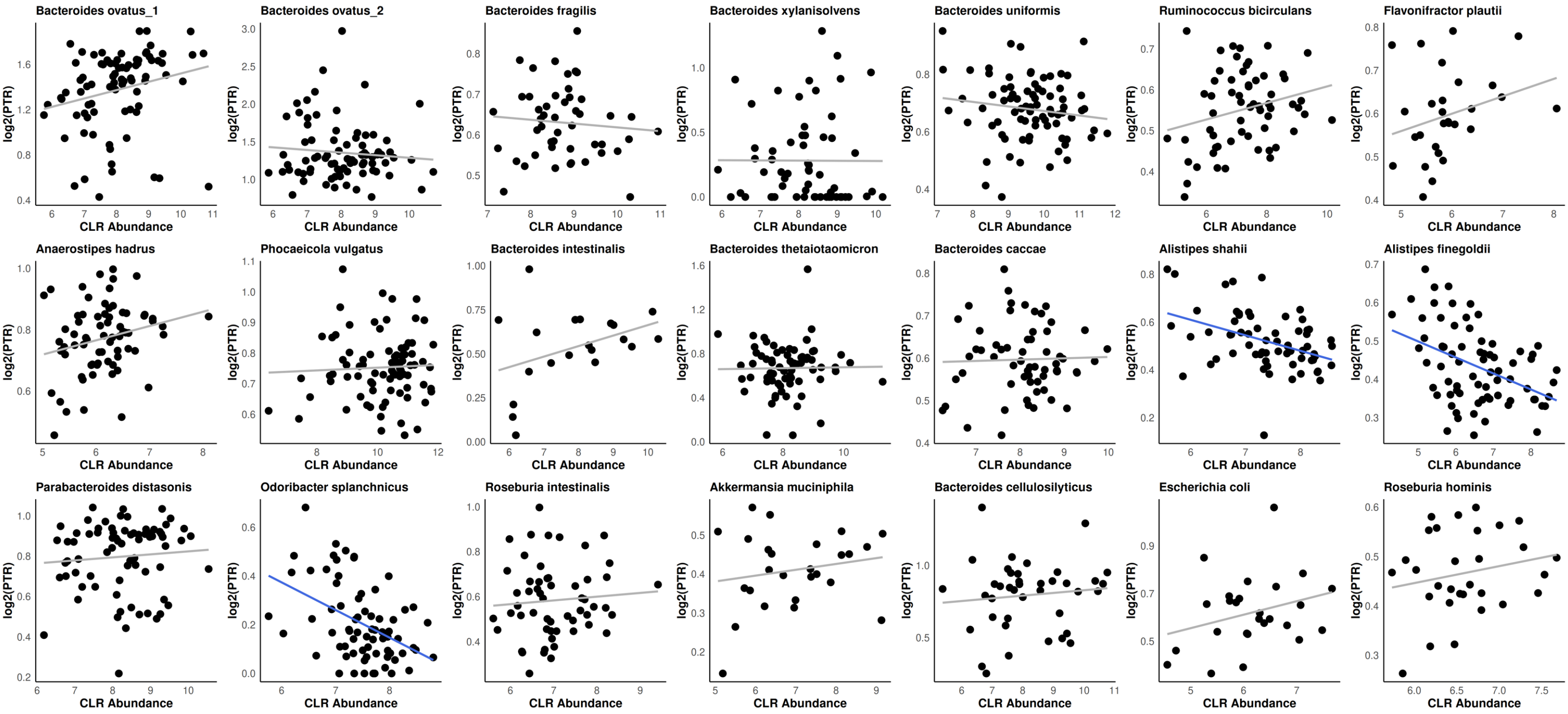

Figure S3. Cross-sectional relationships between average abundance and $\log_2$(PTR) for abundant taxa at the species level across all BIO-ML donors. Abundant taxa that are present in at least 20 out of 78 donors were selected to evaluate the cross-sectional relationship between CLR abundance and $\log_2$(PTR). CLR abundance and $\log_2$(PTR) for taxa that were captured in more than one sampling time point were averaged such that each point in the plot represents one donor. Gray trend lines show no significant correlations and blue trend lines represent significant negative correlations (linear regression, FDR adjusted $p < 0.05$). Alistipes shahii: FDR adjusted $p = 0.00494$; Alistipes finegoldii: FDR adjusted $p = 0.00242$; Odoribacter splanchnicus: FDR adjusted $p = 0.00217$.

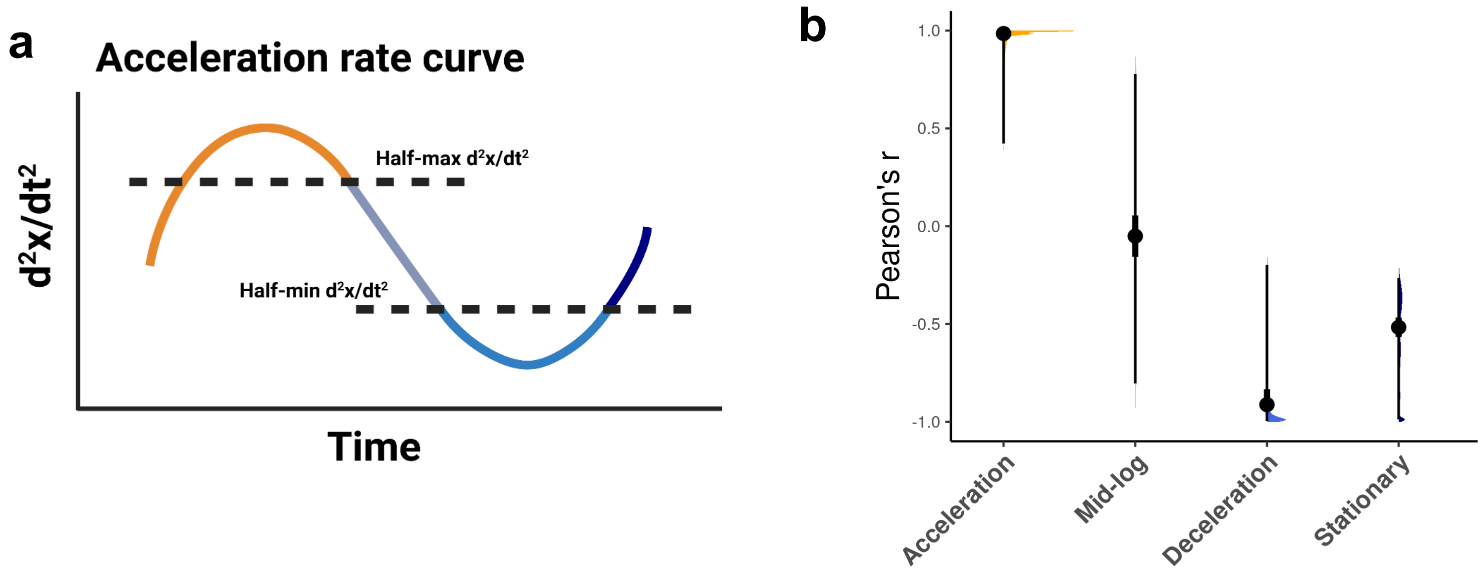

Figure S4. Definition of major growth phases using the stochastic logistic growth model. A. The half-maximum of the peak and half-minimum of the trough of the second derivative of abundance were used to define growth phases across model parameterizations. **B.** Pearson r values between abundances and growth rates in the three growth phase categories obtained from combined sLGE simulation results across a range of growth rates (r = 1-3), carrying capacities (k = 10-1000), and noise levels (n = 0.001-1).

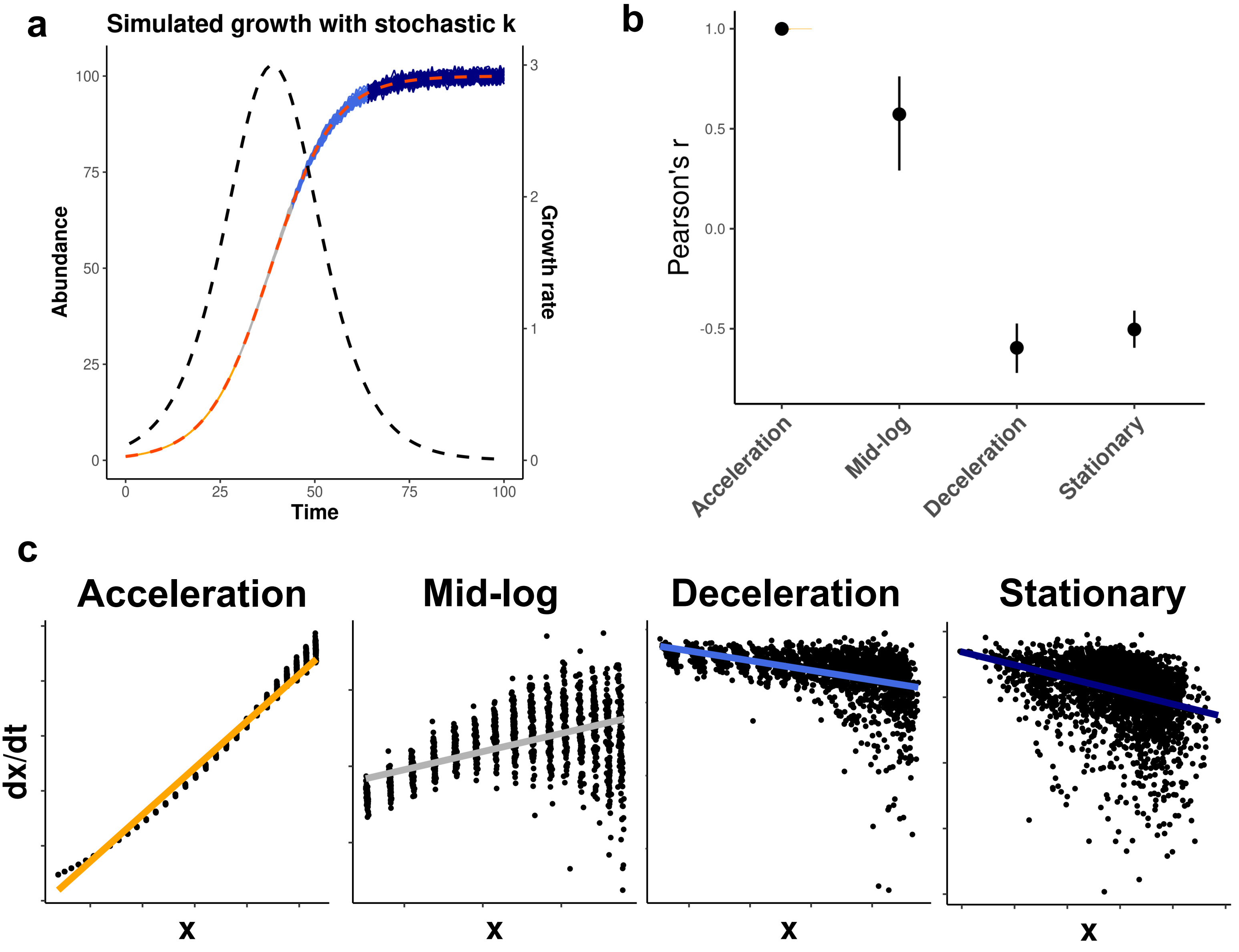

Figure S5. Relationship between growth rate and abundance across the major growth phases, simulated using the logistic growth model with stochastically varying carrying capacities. A. Stochastic logistic growth curves with growth rate (r) = 1.2, carrying capacity (k) = 100, and noise level (n) = 0.1 applied to k across 100 iterations. Major growth phase groups in orange (acceleration), gray (mid-log), blue (deceleration), and navy (stationary). **B.** Pearson r values between abundances and growth rates in each of the four growth phase windows across variable model parameterizations (r = 1-3, k = 10-1000) and a fixed noise level (σ = 0.1). **C.** Scatter plots in log scale showing relationships between abundance and growth rate across the four growth phase regions defined in panel A.

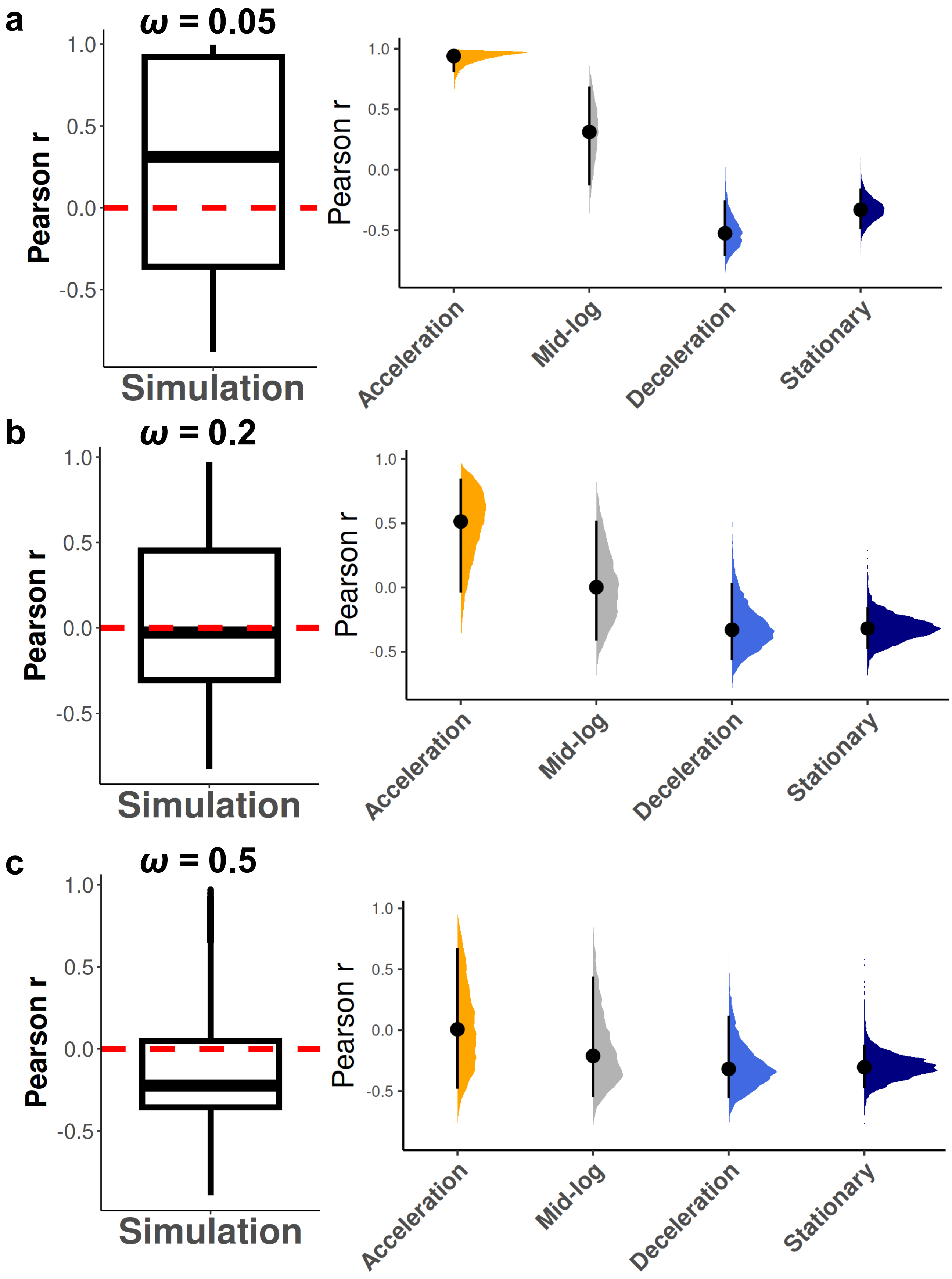
a
ω = 0.05
Pearson r
1.0
0.5
0.0
-0.5
Simulation
Acceleration
Mid-log
Deceleration
Stationary
b
ω = 0.2
Pearson r
Simulation
Acceleration
Mid-log
Deceleration
Stationary
c
ω = 0.5
Pearson r
Simulation
Acceleration
Mid-log
Deceleration
Stationary

Figure S6. Changes in the relationship between growth phase and abundance in the stochastic logistic growth equation with varying noise levels. Logistic growth simulation with growth rate (r) = 1.2 and carrying capacity (k) = 100. Noise (ω) was incorporated in the simulation from ω = 0.05 (**A**) and ω = 0.2 (**B**) to ω = 0.5 (**C**). Simulation was performed with 100 iterations. Left panels in each figure show the range of Pearson r values comparing growth rate and abundance for all growth phases combined (i.e. entire simulation). Right panels in each figure show Pearson r values between growth rate and abundance in each major growth phase. Major growth phase groups in orange (acceleration), gray (mid-log), blue (deceleration), and navy (stationary).

Donor ae

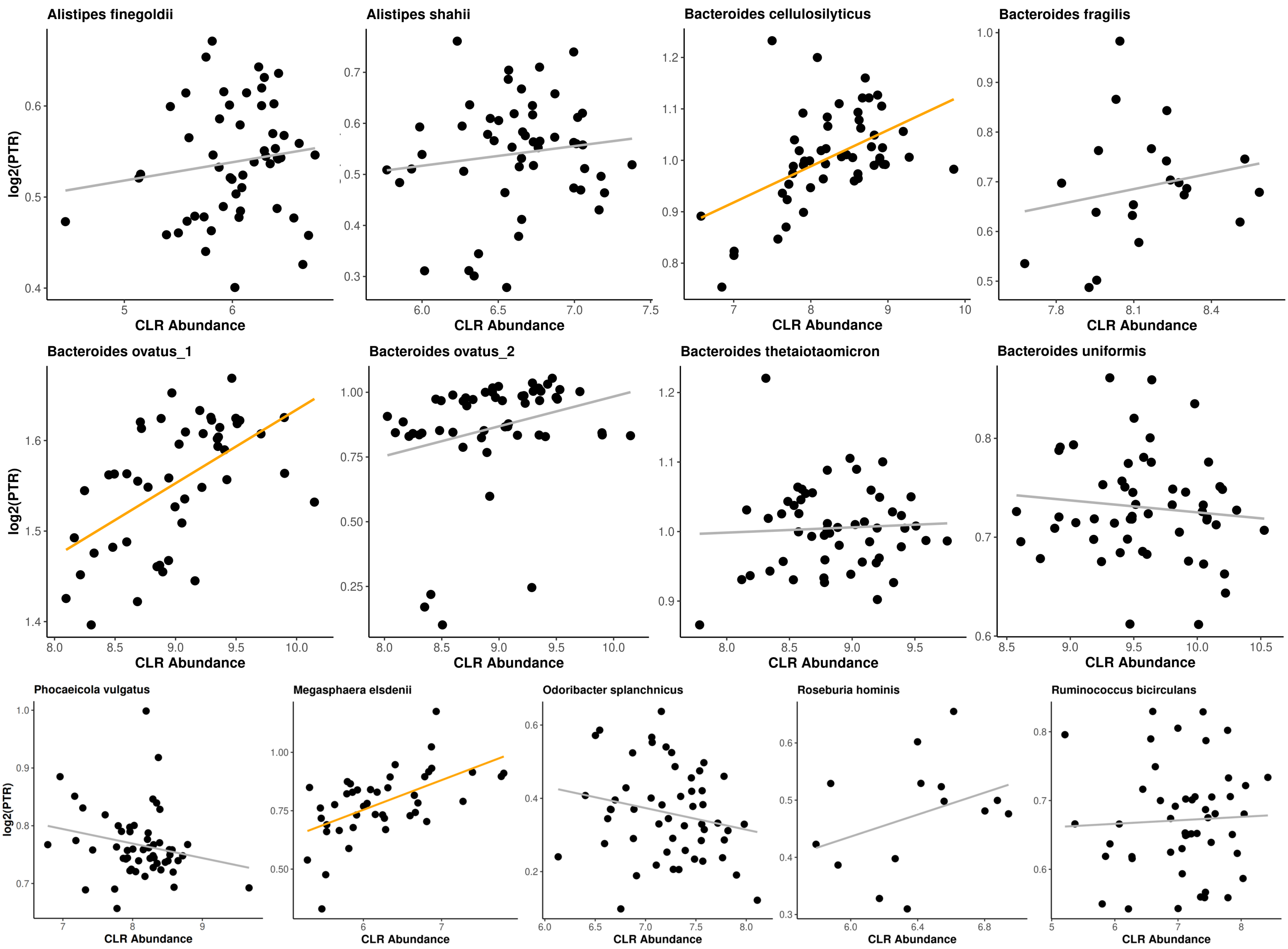

Figure S7. Relationships between abundance and $\log_2$(PTR) for abundant taxa in donor ae. Abundant taxa with relatively dense longitudinal PTR and abundance data (at least 5 matched data points; time differences between adjacent samples less than three days) were selected for analysis. Gray trend lines show no significant correlations, orange trend lines indicate significant positive correlations and blue trend lines represent significant negative correlations (linear regression, FDR-adjusted $p < 0.05$). *Bacteroides cellulosilyticus*: FDR adjusted $p = 7.525e^{-04}$; *Bacteroides ovatus_1*: FDR adjusted $p = 4.943e^{-04}$; *Megasphaera elsdenii*: FDR adjusted $p = 5.091e^{-04}$.

Donor am

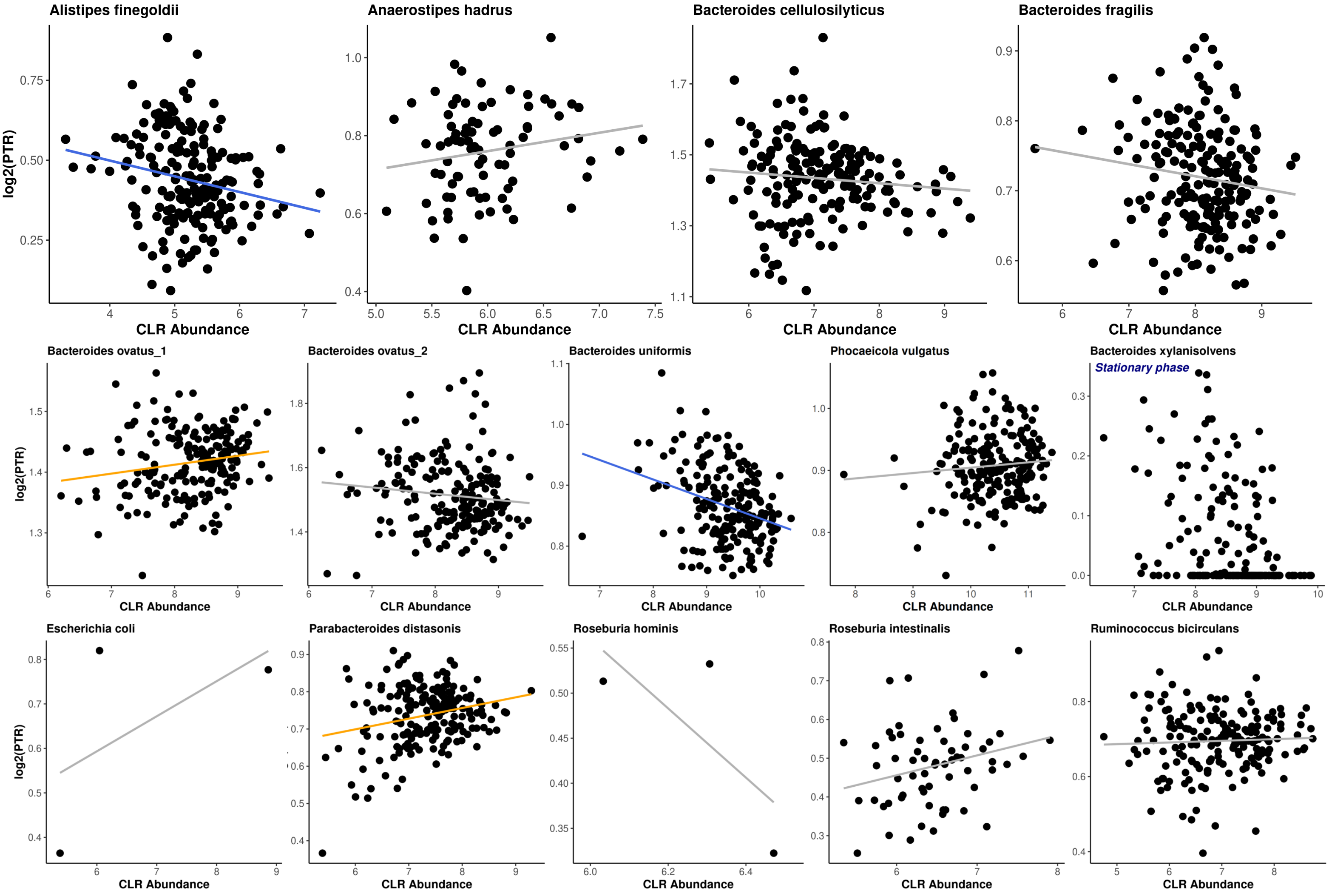

Figure S8. Relationships between abundance and log_2(PTR) for abundant taxa in donor am. Abundant taxa with relatively dense longitudinal log_2(PTR) and abundance data (at least 5 matched data points; time differences between adjacent samples less than three days) were selected for analysis. Gray trend lines show no significant correlations, orange trend lines indicate significant positive correlations and blue trend lines represent significant negative correlations (linear regression, FDR-adjusted $p < 0.05$). *Alistipes finegoldii*: FDR adjusted $p = 1.341e^{-02}$; *Bacteroides ovatus_1*: FDR adjusted $p = 3.166e^{-02}$; *Bacteroides uniformis*: FDR adjusted $p = 5.539e^{-05}$; *Parabacteroides distasonis*: FDR adjusted $p = 2.852e^{-03}$.

Donor an

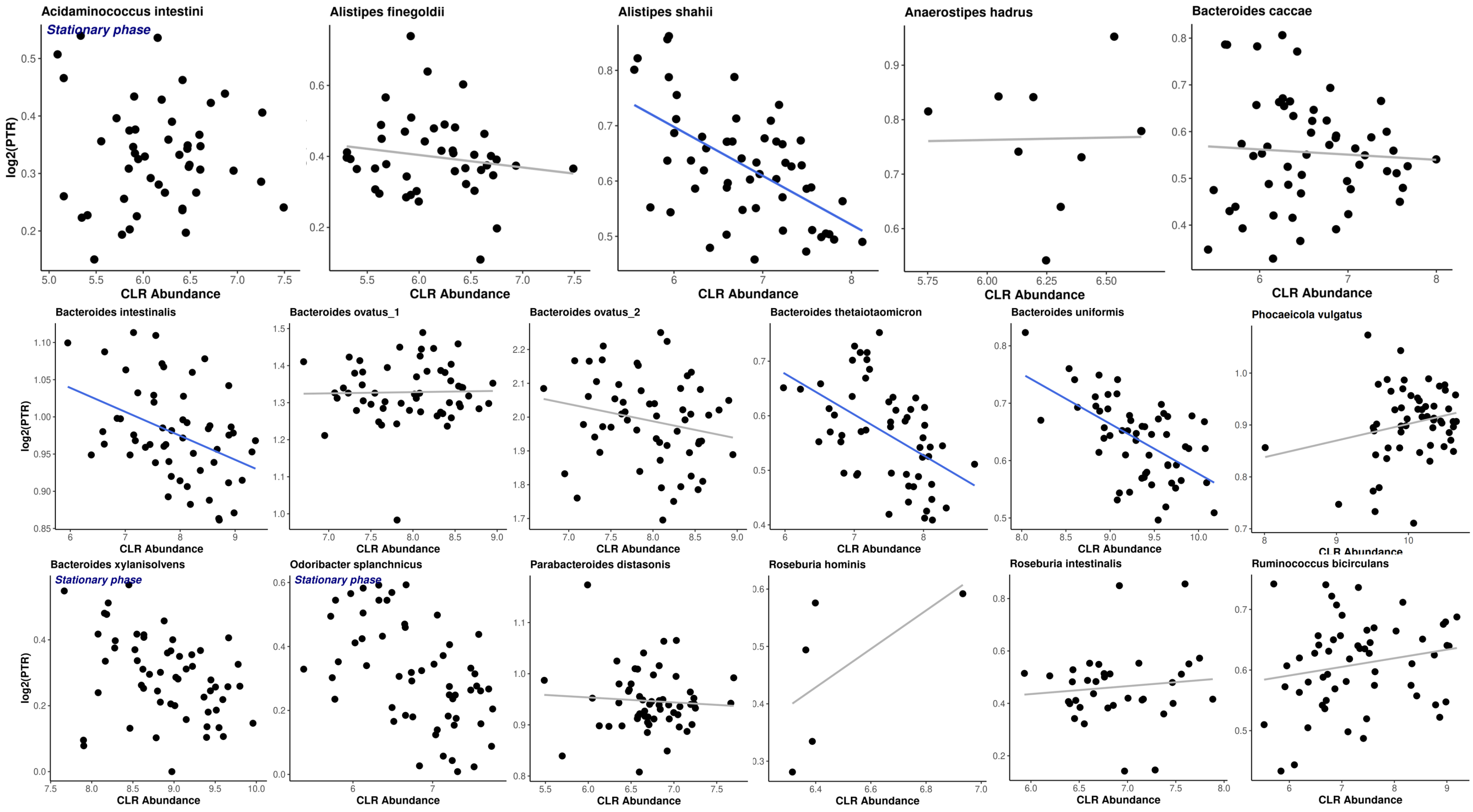

Figure S9. Relationships between abundance and log2(PTR) for abundant taxa in donor an. Abundant taxa with relatively dense longitudinal $\log_2$(PTR) and abundance data (at least 5 matched data points; time differences between adjacent samples less than three days) were selected for analysis. Gray trend lines show no significant correlations, orange trend lines indicate significant positive correlations and blue trend lines represent significant negative correlations (linear regression, FDR-adjusted $p < 0.05$). *Alistipes shahii*: FDR adjusted $p = 5.871e^{-05}$; *Bacteroides intestinalis*: FDR adjusted $p = 6.684e^{-03}$; *Bacteroides thetaiotaomicron*: FDR adjusted $p = 5.60e^{-04}$; *Bacteroides uniformis*: FDR adjusted $p = 5.871e^{-05}$.

Donor ao

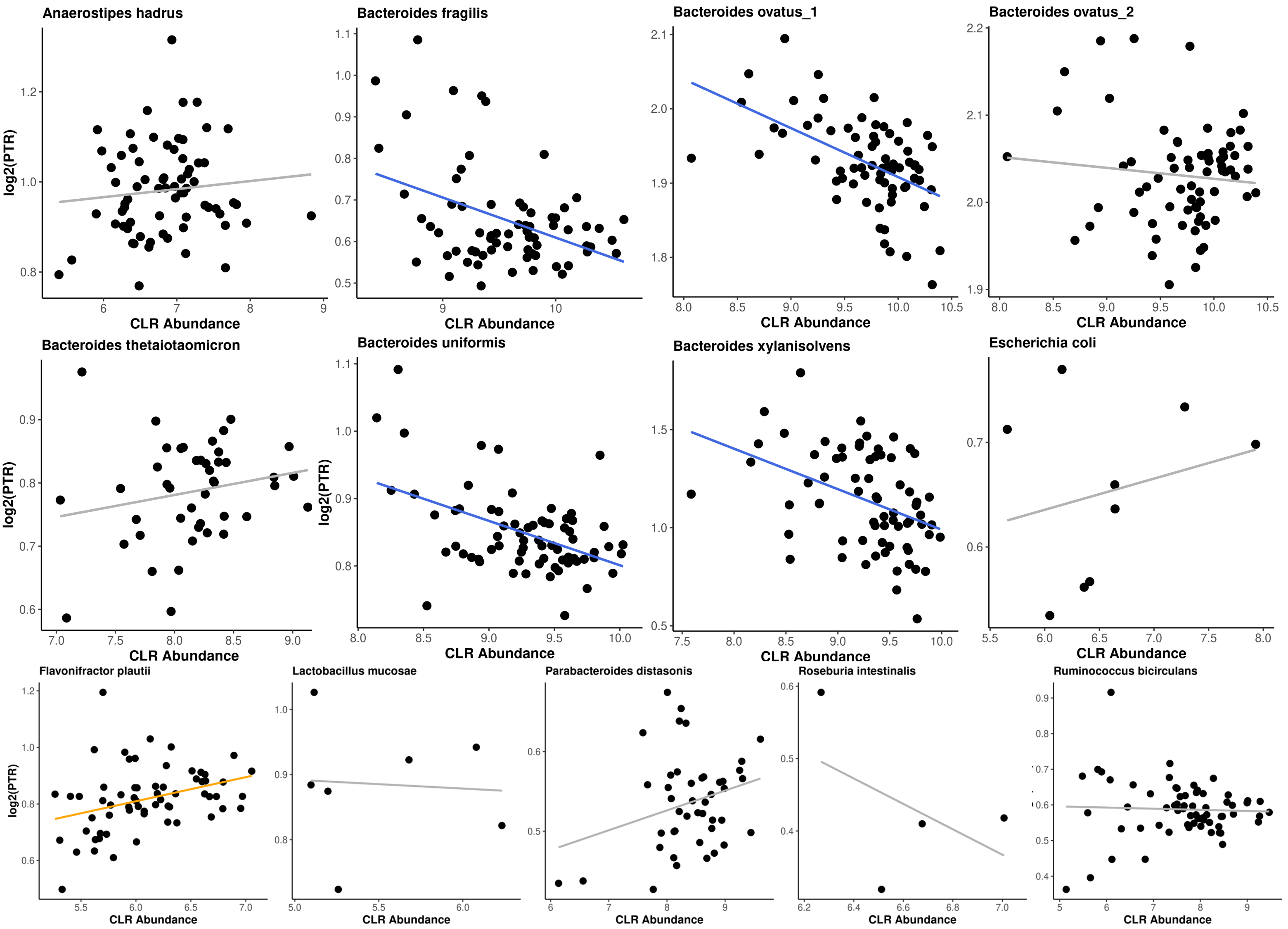

Figure S10. Relationships between abundance and $\log_2$(PTR) for individual taxon in donor ao. Abundant taxa with relatively dense longitudinal $\log_2$(PTR) and abundance data (at least 5 matched data points; time differences between adjacent samples less than three days) were selected for analysis. Gray trend lines show no significant correlations, orange trend lines indicate significant positive correlations and blue trend lines represent significant negative correlations (linear regression, FDR-adjusted $p < 0.05$). *Bacteroides fragilis*: FDR adjusted $p = 1.677e^{-03}$; *Bacteroides ovatus_1*: FDR adjusted $p = 5.415e^{-05}$; *Bacteroides uniformis*: FDR adjusted $p = 2.098e^{-04}$; *Bacteroides xylanisolvens*: FDR adjusted $p = 1.677e^{-03}$; *Flavonifractor plautii*: FDR adjusted $p = 1.366e^{-02}$.

REFERENCES

1. David, L. A. et al. Host lifestyle affects human microbiota on daily timescales. Genome Biol. 15, R89 (2014).
