# Supplementary material for: Growth phase estimation for abundant bacterial populations sampled longitudinally from human stool metagenomes
Source: Nat Commun. 2023 Sep 14;14:5682. doi: 10.1038/s41467-023-41424-1 (PMC10502120; doi:10.1038/s41467-023-41424-1)
Supplement: Supplementary file 3 — Reporting Summary [file 41467_2023_41424_MOESM3_ESM.pdf]

nature portfolio

Corresponding author(s): Sean M. Gibbons

Last updated by author(s): Jul 20, 2023

Reporting Summary

Nature Portfolio wishes to improve the reproducibility of the work that we publish. This form provides structure for consistency and transparency in reporting. For further information on Nature Portfolio policies, see our Editorial Policies and the Editorial Policy Checklist.

Statistics

For all statistical analyses, confirm that the following items are present in the figure legend, table legend, main text, or Methods section.

n/a	Confirmed	
☐	☒	The exact sample size (n) for each experimental group/condition, given as a discrete number and unit of measurement
☐	☒	A statement on whether measurements were taken from distinct samples or whether the same sample was measured repeatedly
☐	☒	The statistical test(s) used AND whether they are one- or two-sided<br>*Only common tests should be described solely by name; describe more complex techniques in the Methods section.*
☐	☒	A description of all covariates tested
☐	☒	A description of any assumptions or corrections, such as tests of normality and adjustment for multiple comparisons
☐	☒	A full description of the statistical parameters including central tendency (e.g. means) or other basic estimates (e.g. regression coefficient) AND variation (e.g. standard deviation) or associated estimates of uncertainty (e.g. confidence intervals)
☐	☒	For null hypothesis testing, the test statistic (e.g. F, t, r) with confidence intervals, effect sizes, degrees of freedom and P value noted<br>*Give P values as exact values whenever suitable.*
☒	☐	For Bayesian analysis, information on the choice of priors and Markov chain Monte Carlo settings
☒	☐	For hierarchical and complex designs, identification of the appropriate level for tests and full reporting of outcomes
☒	☐	Estimates of effect sizes (e.g. Cohen's d, Pearson's r), indicating how they were calculated

Our web collection on statistics for biologists contains articles on many of the points above.

Software and code

Policy information about availability of computer code

Data collection	All human gut metagenomic data analyzed in this study was sourced from the published BIO-ML database (https://pubmed.ncbi.nlm.nih.gov/31477907/). Specifically, raw data was downloaded from SRA SAMN11950000–SAMN11950562 under BioProject PRJNA544527. The raw shotgun sequencing data from the in vitro experiment are publicly available under the SRA BioProject PRJNA942341.
Data analysis	Nextflow pipelines implementing the processing of metagenomic shotgun sequencing data from raw reads to taxonomic abundance matrices and PTR estimates can be found at https://github.com/Gibbons-Lab/pipelines/ (metagenomics pipelines). R scripts and code used to analyze the data, run the sLGE simulations, and produce the figures in the manuscript have been deposited at https://github.com/Gibbons-Lab/human-microbiome-time-series-growth-phase-estimation.

For manuscripts utilizing custom algorithms or software that are central to the research but not yet described in published literature, software must be made available to editors and reviewers. We strongly encourage code deposition in a community repository (e.g. GitHub). See the Nature Portfolio guidelines for submitting code & software for further information.

Data

Policy information about availability of data

All manuscripts must include a data availability statement. This statement should provide the following information, where applicable:

- Accession codes, unique identifiers, or web links for publicly available datasets
- A description of any restrictions on data availability
- For clinical datasets or third party data, please ensure that the statement adheres to our policy

Nextflow pipelines implementing the processing of metagenomic shotgun sequencing data from raw reads to taxonomic abundance matrices and PTR estimates can be found at https://github.com/Gibbons-Lab/pipelines/ (metagenomics pipelines). R scripts and code used to analyze the data, run the sLGE simulations, and

produce the figures in the manuscript have been deposited at https://github.com/Gibbons-Lab/human-microbiome-time-series-growth-phase-estimation. All human gut metagenomic data analyzed in this study was sourced from the published BIO-ML database (https://pubmed.ncbi.nlm.nih.gov/31477907/). Specifically, raw data was downloaded from SRA SAMN11950000–SAMN11950562 under BioProject PRJNA544527. The raw shotgun sequencing data from the in vitro experiment are publicly available under the SRA BioProject PRJNA942341.

Field-specific reporting

Please select the one below that is the best fit for your research. If you are not sure, read the appropriate sections before making your selection.

- [x] Life sciences
- [ ] Behavioural & social sciences
- [ ] Ecological, evolutionary & environmental sciences

For a reference copy of the document with all sections, see nature.com/documents/nr-reporting-summary-flat.pdf

Life sciences study design

All studies must disclose on these points even when the disclosure is negative.

Sample size	We analyzed data from four densely-sampled human gut metagenomic time series (> 50 time points per individual) and single time points from 84 individuals. We also sequenced 80 time point samples from an in vitro growth curve of Escherichia coli.
Data exclusions	We selected BIO-ML donors with >50 time points for longitudinal analyses (N=4) and we analyzed individual samples from the 84 BIO-ML donors for cross-sectional analyses.
Replication	We predict statistical associations between population sizes and growth rates from a simple growth model (i.e., stochastic logistic growth) that vary across growth phases. We validate our model predictions using real-world data from four human gut metagenomic time series and with an in vitro growth experiment. Long, densely sampled metagenomic time series from flow-through ecosystems are rare. BIO-ML provided us with a unique opportunity to test our model predictions. Future work should focus on generating additional data sets that can be used to test our model predictions.
Randomization	NA
Blinding	NA

Reporting for specific materials, systems and methods

We require information from authors about some types of materials, experimental systems and methods used in many studies. Here, indicate whether each material, system or method listed is relevant to your study. If you are not sure if a list item applies to your research, read the appropriate section before selecting a response.

Materials & experimental systems

n/a	Involved in the study	
[x]	[ ]	Antibodies
[x]	[ ]	Eukaryotic cell lines
[x]	[ ]	Palaeontology and archaeology
[x]	[ ]	Animals and other organisms
[x]	[ ]	Human research participants
[x]	[ ]	Clinical data
[x]	[ ]	Dual use research of concern

Methods

n/a	Involved in the study	
[x]	[ ]	ChIP-seq
[x]	[ ]	Flow cytometry
[x]	[ ]	MRI-based neuroimaging
